# Apoptotic-like programed cell death in fungi: the benefits in filamentous species

**DOI:** 10.3389/fonc.2012.00097

**Published:** 2012-08-07

**Authors:** Neta Shlezinger, Nir Goldfinger, Amir Sharon

**Affiliations:** Department of Molecular Biology and Ecology of Plants, Tel Aviv University,Tel Aviv, Israel

**Keywords:** apoptosis, botrytis, fungi, PCD, *Saccharomyces*

## Abstract

Studies conducted in the early 1990s showed for the first time that *Saccharomyces cerevisiae* can undergo cell death with hallmarks of animal apoptosis. These findings came as a surprise, since suicide machinery was unexpected in unicellular organisms. Today, apoptosis in yeast is well-documented. Apoptotic death of yeast cells has been described under various conditions and *S. cerevisiae* homologs of human apoptotic genes have been identified and characterized. These studies also revealed fundamental differences between yeast and animal apoptosis; in *S. cerevisiae* apoptosis is mainly associated with aging and stress adaptation, unlike animal apoptosis, which is essential for proper development. Further, many apoptosis regulatory genes are either missing, or highly divergent in *S. cerevisiae*. Therefore, in this review we will use the term apoptosis-like programed cell death (PCD) instead of apoptosis. Despite these significant differences, *S. cerevisiae* has been instrumental in promoting the study of heterologous apoptotic proteins, particularly from human. Work in fungi other than *S. cerevisiae* revealed differences in the manifestation of PCD in single cell (yeasts) and multicellular (filamentous) species. Such differences may reflect the higher complexity level of filamentous species, and hence the involvement of PCD in a wider range of processes and life styles. It is also expected that differences might be found in the apoptosis apparatus of yeast and filamentous species. In this review we focus on aspects of PCD that are unique or can be better studied in filamentous species. We will highlight the similarities and differences of the PCD machinery between yeast and filamentous species and show the value of using *S. cerevisiae* along with filamentous species to study apoptosis.

## OVERVIEW

Organisms in the fungal kingdom can be separated into two distinct morphotypes: unicellular (yeasts) and multicellular (filamentous), with some species having a dimorphic appearance. Although this separation does not have a phylogenetic basis, the different in morphology also extends to the molecular level. Yeasts are the better studied group due to their long association with human civilization and ease of use; the combination of eukaryotic single cell type, genetic tractability, and the ability to easily quantify cell populations, make yeasts excellent research systems. In particular, the baker’s yeast *Saccharomyces cerevisiae* has been developed as an eukaryotic model to study cellular and developmental processes, including programed cell death (PCD). Originally, *S. cerevisiae* was used as a system to evaluate and search for human apoptotic proteins (Sato et al., 1994; Xu and Reed, 1998). These studies lead to the discovery and study of PCD in *S. cerevisiae* (Madeo et al., 1997). Research of PCD was later extended to additional fungi, including filamentous species. These studies revealed substantial variability in the regulation and manifestation of PCD in different species, and especially between *S. cerevisiae* and filamentous fungi. Most significantly, processes such as multicellular development and pathogenicity, in which PCD may play a significant role, cannot be studied in *S. cerevisiae*. We will compare the current status of knowledge on PCD in *S. cerevisiae* and filamentous species, and highlight the advantages of using *S. cerevisiae* along with filamentous species in the study of PCD.

## PCD IN *S. CEREVISIAE*

In metazoans there are two major apoptotic pathways: the extrinsic pathway, composed of a so called death receptors and ligands of the TNF family, and the intrinsic pathway culminating in mitochondrial outer membrane permeability. In mammals the extrinsic pathway is mediated by the death-inducing signaling complex (DISC), which contains a death receptor trimer, FADD adaptor proteins and caspases 8 and 10. The intrinsic pathway is initiated by the release of cytochrome *c* from the mitochondria following apoptotic stimuli, which along with Apaf-1 and procaspase 9 form a heptameric complex known as the apoptosome (Mace and Riedl, 2010). Pro- and anti-apoptotic members of the Bcl-2 family of proteins, which function upstream of or at the mitochondria membrane, are central regulators of PCD in animals (Chipuk et al., 2010).

Programed cell death is induced in yeast by a variety of triggers and is accompanied by most if not all the typical characteristics of animal apoptosis (Xu and Reed, 1998; Rockenfeller and Madeo, 2008; Schmitt and Reiter, 2008; Carmona-Gutierrez et al., 2010). Nevertheless, the yeast apparatus bears significant differences compared to apoptotic apparatus in animals. Most significantly, the entire extrinsic pathway is not found in fungi. Furthermore, important regulators of the intrinsic pathway, including Bcl-2 proteins, P-53, FLIP, poly ADP-ribose polymerase (PARP), and even caspases do not have clear homologs in *S. cerevisiae*. Interestingly, homologs of some of the proteins that are not present in *S. cerevisiae* can be found in filamentous species (see below). Such differences at the molecular level are indicative of significant functional differences and should be taken into consideration when comparing fungal and animal PCD.

The most highly represented apoptosis-related proteins found in yeast are mitochondria-associated proteins. In particular, a significant portion of the apoptosis-promoting, mitochondria-secreted proteins have been identified, including homologs of genes encoding for cytochrome *c*, the endonucleases apoptosis-inducing factor (AIF) and EndoG, and the IAP-inhibiting serine protease Omi/HrtA2. In addition, several orthologs of non-mitochondrial proteins have been analyzed (for a review, see Carmona-Gutierrez et al., 2010). Interestingly, the only known executor of apoptosis in *S. cerevisiae* is the metacaspase Yca1/Mca1, which mediates the final stages of cell death following a wide range of stimuli (Madeo et al., 2009). Likewise, Bir1p, a class II IAP protein and homolog of human survivin, is the only known inhibitor of apoptosis in yeasts (Owsianowski et al., 2008). In addition to homologs of apoptosis proteins, a number of mitochondria proteins that are involved in mitochondria fusion, fission, and homeostasis also affect yeast apoptosis (Fröhlich et al., 2007). Deletion of the *S. cerevisiae* dynamin related protein Dnm1p, which is responsible for mitochondrial fission caused elongation of mitochondria and subsequent increase of life (Scheckhuber et al., 2007; Carmona-Gutierrez et al., 2010). Mutants in Fis1p, an anchor protein for Dnm1p, increased sensitivity of the yeast cells to apoptosis, probably due to selection for a *whi2* mutation (Teng et al., 2011). The microtubule and mitochondria interacting protein Mmi1p, an ortholog of human Tctp, shuttles from the cytoplasm to mitochondria upon an apoptosis stimulus and promotes PCD in yeast cells (Rinnerthaler et al., 2006).

Despite the absence of a significant portion of the animal apoptotic network, *S. cerevisiae* has proven a viable system to study human apoptosis. These studies stem from the first observation that expression of human Bcl-2 pro- and anti-apoptotic proteins promote or suppress PCD in yeast cells, respectively (Sato et al., 1994; Xu and Reed, 1998). Nevertheless, as a single cell organism, the results obtained in *S. cerevisiae* are limited to cellular processes and relevance to situations in animals is not always clear; primarily, multicell level development cannot be studied in *S. cerevisiae*. In addition, certain PCD-related processes such as aging and autophagy might be significantly different in the context of multicellular organisms compared with unicellular organisms. In these instances filamentous fungi might be useful in complementing and augmenting the results obtained in yeasts.

## PCD IN FILAMENTOUS FUNGI

Filamentous fungi combine the genetic simplicity and short life cycle of yeast with the morphological complexity of multicellular organism. They typically form a network of interconnected hyphae, which are defined as “colonies” that grow by hyphal tip extension, branching, and fusion. In higher fungi (Ascomycotina and Basidiomycotina, subkingdom Dikarya), the septa along the hyphae are incomplete, leaving a pore through which cytoplasm and organelles can move (Glass and Fleissner, 2006). PCD has been observed in higher fungi during sexual and asexual development, for example during gills formation in mushrooms or formation of sclerotia in some Ascomycetes (Georgiou et al., 2006). This type of coordinated cell death echoes developmental PCD in higher eukaryotes. In addition, and similar to the situation in yeasts, PCD in filamentous fungi is also associated with stress adaptation, spore formation, antagonistic interactions, and aging (Sharon et al., 2009). However, some aspects of fungal PCD are significantly different between single cell and filamentous species. These differences might stem from the different lifestyles of single cell and multicellular organisms. In addition to differences due to unicellular and multicellular organization, there are processes related to PCD that either cannot be analyzed in *S. cerevisiae*, e.g., pathogenicity, or are significantly different in multicellular species. The use of filamentous species in these cases is of special importance.

### PCD AND AGING

Aging is a process of progressive decline in the ability to withstand stress, damage, and disease. Aging processes have been extensively studied in various model organisms including *S. cerevisiae*. In addition, the filamentous fungus *Podospora anserina* has been used as a model to study aging in multicellular eukaryote (Osiewacz, 2002, 2011). In fact, study of aging in *P. anserina* started already in the 1950s, and the connection of mitochondria and aging was demonstrated for the first time in this fungus (Rizet, 1953). In *P. anserina*, senescence is characterized by an age related decrease in mycelium growth rate, reduction in formation of aerial hyphae, increased pigmentation, and eventual death of peripheral hyphae (Albert and Sellem, 2002; Scheckhuber and Osiewacz, 2008). At the microscopic level, the peripheral hyphae show abnormal branching and swelling. In wild-type isolates of *P. anserina*, aging is correlated with accumulation of mutated mtDNA leading to mitochondrial genome instability (Stahl et al., 1978; Kuck et al., 1985; Osiewacz and Borghouts, 2000; Albert and Sellem, 2002). The instability of the mitochondria genome correlates with appearance and accumulation of a 2.5-kb DNA fragments that correspond to an integral part of the 95-kb mtDNA and to the first intron (pl-intron) of the *PaCOX1* gene, the first subunit of cytochrome *c* oxidase (Cox) in the respiratory chain. Strains selected for increased lifespan were found to be deficient in Cox activity due to deletion of the first exon of the *PaCOX1* gene. Deletion of *PaCOX5* (encoding subunit V of Cox) led to severe decrease in growth rate, along with decreased ROS production, drastic reduction in the rearrangement of mtDNA, and a 30-fold increased lifespan of the fungus (Dufour et al., 2000). Mutants with deletions in genes encoding other Cox subunits had a similar phenotype (Lorin et al., 2006). In these mutants, respiration was carried out via alternative oxidase (Aox)-dependent pathways, an enzyme of the inner mitochondrial membrane. Genetic manipulation that restored ROS production to wild-type levels also reversed the amount of mutated mtDNA to wild-type levels and reversed lifespan of the strains to wild-type levels. Deletion of *PaDNM1* forced increased lifespan and reduced sensitivity to the apoptosis-inducing compound etoposide, further demonstrating the central role of mitochondrion-mediated PCD in aging (Scheckhuber et al., 2007). Collectively, these results indicate that increased ROS levels during aging trigger mitochondria-dependent PCD in senescent cultures of *P. anserina*. Deletion of putative AIFs also leads to lifespan extension, providing evidence that aging in *P. anserina* is programed and tightly connected with PCD (Hamann et al., 2007; Brust et al., 2010).

Similar to all other systems, the final stages of PCD in fungi are carried out by cysteine proteases exhibiting caspase activity. At least one, but usually two or three caspase-related genes are found in fungi. While the enzymes encoded by these genes recognize the typical substrates of caspases, the encoded proteins show limited homology to animal caspases. Furthermore, they lack a cas domain, the most significant signature of caspases. It has been proposed that these proteins represent an ancient form of caspases and therefore they were termed metacaspases (Savoldi et al., 2008; Tsiatsiani et al., 2011). A caspase-independent pathway also exists in fungi, which (similar to situation in human) involves homologs of AIF and AIF-homologous mitochondrion-associated inducers of death (AMID; Modjtahedi et al., 2006).

Functional analyses of the metacaspase-dependent and -independent pathways were conducted by deletion of either the metacaspases or AIF members in *P. anserina*. Deletion of either of the two putative metacaspases, *PaMCA1* and *PaMCA2*, in *P. anserina* reduced sensitivity to PCD-promoting conditions and had a lifespan extending effect on the fungus (Hamann et al., 2007). The AIF family in *P. anserina* includes at least five members that are divided to cytosolic and mitochondria species. Deletion of the mitochondria-residing members, either *PaAIF2* or *PaAMID2*, caused reduced sensitivity to oxidative conditions and extended lifespan of the fungus. In contrast, deletion of the cytosolic isoforms of AIF, *PaAIF1* and *PaAMID1*, had no effect on lifespan and on sensitivity of the fungus to oxidative stress (Brust et al., 2010).

Together, *S. cerevisiae* and *P. anserina* form an excellent system for unraveling the role of mitochondria in aging. Both species are capable of adjusting their metabolism in case of mitochondria dysfunction, but *S. cerevisiae* does not have the Aox pathway, which is used by *P. anserina* to compensate for Cox deficiency. *S. cerevisiae* also lacks complex I of the mitochondria respiratory chain and therefore this complex can only be studied in *P. anserina* (Osiewacz and Scheckhuber, 2006). Likewise, *S. cerevisiae* can grow under anaerobic conditions, and hence is useful in studying processes that might be lethal in strict aerobes such as *P. anserina*.

### PCD AND FUNGAL PATHOGENESIS

During pathogenic interaction both the host and pathogen are exposed to PCD-inducing conditions and compounds (Sharon and Finkelshtein, 2009). Interestingly, all plant pathogenic fungi are filamentous in nature. While not as strict, most human fungal pathogens also are either filamentous or dimorphic. Furthermore, dimorphic pathogenic species, such as the human pathogen *Candida albicans* or the maize pathogen *Ustilago maydis*, switch from a yeast to a filamentous state during transition from a latent to a pathogenic state (Garber and Day, 1985; Lo et al., 1997). Hence, filamentous fungi can be used to study the role of pathogen PCD in plant and animal diseases.

In plants, the manipulation of the host apoptotic response, either enhancement (by necrotrophic pathogens) or suppression (by biotrophic pathogens) of PCD, is a common strategy used by fungi to weaken the host (Sharon and Finkelshtein, 2009). This phenomenon was demonstrated in transgenic plants expressing anti-apoptotic genes, which suppressed PCD and enhanced or reduced plants’ susceptibility to either biotrophic or necrotrophic pathogens, respectively (Dickman et al., 2001; Huckelhoven et al., 2001, 2003; del Pozo and Lam, 2003; Eichmann et al., 2004). A number of studies demonstrated limited necrosis and restricted spreading of the model necrotrophic pathogen *Botrytis cinerea* in plants that over-express anti-apoptotic genes or in hypersensitive response (HR)-deficient mutant plants that do not produce ROS, whereas accelerated cell death mutant plants are more susceptible to this pathogen (Govrin and Levine, 2000; Imani et al., 2006; Van Baarlen et al., 2007). Dihydrosphingosine-induced cell death was shown to mediate phytotoxicity of AAL toxin. This toxin is produced by the necrotrophic pathogen *Alternaria alternata* and belongs to a class of host-selective fungal mycotoxins that are structurally related to sphinganine, a precursor in plant sphingolipid biosynthesis. AAL toxin kills the cells of sensitive host plants by inducing PCD (Brandwagt et al., 2000). Administration of AAL toxin to sensitive tissues blocks sphingolipid biosynthesis and leads to accumulation of dihydrosphingosine. AAL-insensitive plants contain the *ASC-1* resistance gene, a homolog of the yeast longevity assurance gene (*LAC1*). Asc1p modifies sphingolipid metabolism in AAL-treated cells, thereby preventing accumulation of dihydrosphingosine and induction of apoptosis (Brandwagt et al., 2000; Spassieva et al., 2002).

Several studies documented fungal cell death during infection and showed that it was essential for completion of pathogenic life cycle (Howard et al., 1991; Thines et al., 2000; Veneault-Fourrey et al., 2006). In contrast, Barhoom and Sharon (2007) reported on hyper virulence of a cell death-protected *Colletotrichum gloeosporioides* strain, over-expressing human Bcl-2. These studies hint to a link between fungal PCD and disease. Early studies showed that some plant compounds, for example the tobacco pathogenesis related protein osmotin, can induce PCD in *S. cerevisiae* (Narasimhan et al., 2001). Additional antifungal peptides from other organisms were found, which can induce PCD in different fungi (Ramsdale, 2008), however the relevance of these results to pathogenesis remains unclear. More recent studies provided new and more direct evidences that plant defense compounds induce PCD in fungi during plant colonization. The saponin α-tomatine, a sesquiterpene glycoside produced by tomato, has antifungal activity. Initially, α-tomatine was considered to promote fungal death by disruption of membrane integrity (Friedman, 2002). A more recent study showed that α-tomatine induces PCD in the plant pathogen *Fusarium oxysporum*. Moreover, PCD was found necessary for antifungal activity of the compound (Ito et al., 2007). Treatment with either ROS scavengers (ascorbic acid and dimethylthiourea) or a caspase inhibitor (Z-VAD-FMK) reduced fungal cell death in a dose-dependent manner, suggesting that α-tomatine-induced cell death in *F. oxysporum* is ROS and caspase-dependent. In addition, the fungicidal action of α-tomatine was suppressed by the mitochondrial electron transport inhibitor oligomycin, suggesting a role for mitochondria in the process.

A more recent example demonstrated the role of PCD in pathogenicity of *B. cinerea*. Camalexin, the major phytoalexin produced in *Arabidopsis*, belongs to a group of secondary metabolites with anti-microbial activity that are produced in plants upon microbial attack (collectively called phytoalexins) and form a line of defense against potential pathogens (Kliebenstein et al., 2005; Lazniewska et al., 2010). Similar to other phytoalexins, camalexin has growth inhibiting activity against a wide range of microorganisms (Ferrari et al., 2003; Kliebenstein et al., 2005; Rowe et al., 2010). Micromolar concentrations of camalexin were found to induce PCD in *B. cinerea*, but at higher concentrations of camalexin the apoptotic markers were reduced, indicating that at these concentrations necrotic cell death was induced (Finkelshtein et al., 2011; Shlezinger et al., 2011b). Similar results were also observed following treatment of *B. cinerea* with hexanoic acid, another plant defense compound (Finkelshtein et al., 2011). These results suggest that when exposed to plant defense molecules during the early phase of infection, *B. cinerea* might be subjected to host-induced PCD. In this event, fungal anti-apoptotic machinery might be necessary for survival and pathogenicity. In order to investigate this possibility, Shlezinger et al. (2011b) tested the role of *B. cinerea* anti-apoptotic BcBir1 protein in disease. This study revealed that following germination and formation of first contact with the plant, the fungus undergoes massive PCD [between 30 and 48 h post-inoculation (PI)], and then fully recovers at 72 h PI, when spreading lesions start to develop. PCD-modified strains were produced by manipulation of the *BcBIR1* gene; over-expression strains were less sensitive, and knockdown strains were hypersensitive to apoptosis induction, respectively. Plant infection assays showed enhanced and reduced virulence of the *BcBIR1* over-expression and knockdown strains, respectively. Importantly, the levels of PCD in *BcBIR1* over-expression strains was markedly reduced between 30 and 48 h PI compared to almost complete elimination of the wild-type cells at this time point. In contrast, in the knockdown strain there was early and intense PCD and it remained high also at 72 h PI, when the wild-type cells showed complete recovery. On *Arabidopsis thaliana* mutant plants that are impaired in defense responses and are hypersensitive to *B. cinerea*, PCD levels were reduce in all strains, confirming that the amount of fungal PCD is negatively correlated with plant susceptibility to the fungus. Specifically, the phytoalexin-deficient *pad3* mutant, which does not produce camalexin, was highly susceptible to *B. cinerea*, and disease was produced on this line also following infection with the *Bcbir1* knockdown strain. As pointed out, camalexin induced PCD in *B. cinerea* wild-type strain *in vitro*. In accordance with the PCD-promoting effect of camalexin, the *BcBIR1* over-expression and knockdown strains showed reduced or enhanced sensitivity to camalexin, respectively, along with reduced PCD on the *pad3* plants.

### PCD IN CELL–CELL INTERACTIONS: HETEROKARYON INCOMPATIBILITY

In filamentous fungi, vegetative hyphae commonly fuse. These hyphal fusions occur during colony formation as well as between hyphae of different strains as part of parasexual reproduction (Saupe et al., 2000; Glass and Kaneko, 2003; Glass and Dementhon, 2006). The fusion between hyphae from different strains leads to formation of a heterokaryon, a situation in which cells contain nuclei of different genetic background. Specific heterokaryon-incompatibility (HI) loci determine fusion compatibility between hyphae from different strains (Leslie and Zeller, 1996; Glass et al., 2000). When hyphae that are not vegetative compatible fuse, a rapid, localized cell death is activated that specifically kills the fusion cell and prevents heterokaryon formation (Glass and Kaneko, 2003).

In many ways, HI resembles the HR in plants, during which localized PCD prevents pathogen spreading (Lam et al., 2001). Both HI and HR are accompanied by classical apoptotic markers and have been widely studied (del Pozo and Lam, 1998; Jacobson et al., 1998; Glass et al., 2000; Saupe et al., 2000; Marek et al., 2003; Glass and Dementhon, 2006; Paoletti and Clave, 2007; Williams and Dickman, 2008). During HI, the fusion hyphae undergo a series of apoptosis-associated morphological changes, including cytoplasm condensation, vacuolization, and shrinkage of the plasma membrane (Glass and Kaneko, 2003; Marek et al., 2003; Glass and Dementhon, 2006). Nuclear fragmentation and positive TUNEL staining have also been documented. Data from whole genome microarrays of *Neurospora crassa* showed that ROS, phosphatidylinositol and calcium signaling, are all involved in HI and PCD. However, homologs of apoptotic genes, such as caspases (metacaspases) and AIF were not required for HI in *N. crassa* (Hutchison et al., 2009).

Severin and Hyman (2002) showed that in the absence of an appropriate mating partner, exposure of yeast cells to pheromones of the opposite mating type leads to ROS accumulation, DNA degradation, and cell death. It should be noted however that pheromone-induced cell death was observed at pheromone concentrations that were 10-fold higher than physiological concentrations; no cell death was induced by physiological concentrations of the mating pheromone. Unlike the case of yeast pheromones, PCD is a general phenomenon of HI and occurs naturally. The widespread occurrence and high number of HI loci in filamentous fungi argues for their importance. Therefore, HI represents an important process in which PCD plays major role. This system can be used in functional and mechanistic studies of heterologous apoptotic proteins and has several advantages over other systems, including budding yeasts. Mainly, the induction of PCD during HI is very rapid and it does not require application of exogenous substances (Garnjobst and Wilson, 1956; Biella et al., 2002; Glass and Kaneko, 2003; Sbrana et al., 2007). Thus, apoptosis can be studied under natural conditions in a short time period, in contrast to PCD induced by aging or starvation.

### ANTIFUNGAL DRUGS AND PCD

Recognition in the importance of PCD in fungi has led to re-evaluation of the mode of action of leading antifungal drugs. Surprisingly, it was found that a range of well-known antifungal compounds induce PCD in fungi. For many years amphotericin B (AmB) has been the most common drug used to treat fungal infections (Brajtburg et al., 1990). Similar to other polyene antibiotics, AmB has high affinity to sterols, particularly ergosterol. The antifungal activity of AmB was attributed to formation of pores in the cell membrane and hence distortion of the fungal cell integrity (Liao et al., 1999). More recently it was found that AmB induces PCD in fungi, including the human pathogens *C. albicans* and *A. fumigatus* (Phillips et al., 2003; Mousavi and Robson, 2004). Notably, at concentrations of 1 mg/ml AmB or higher, cell death shifted from apoptotic to necrotic, as determined by increased and decrease propidium iodine- and TUNEL-positive cells, respectively. Similar to HI PCD, appearance of apoptotic markers could not be blocked or reduced by caspase inhibitors, nor were any changes recorded in caspase activity, suggesting a caspase-independent process. Additional antifungal drugs of different chemical groups have been reported to induce PCD in fungi, suggesting that induced PCD might be a common mode of action for many antifungal compounds (Ramsdale, 2008). The induction of apoptosis by AmB might be mediated by sphingolipids that are released from the plasma membrane. Sphingolipid metabolism is associated with a wide range of cellular activities, including stress response, apoptosis, inflammation, cell-cycle regulation, and cancer development (Dickson, 1998; Kolesnick and Krönke 1998; Hannun and Luberto, 2000; Hannun et al., 2001). Two major sphingoid bases of fungi – dihydrosphingosine and phosphosphingosine, induced ROS accumulation and cell death with typical markers of apoptosis in *Aspergillus nidulans* (Cheng et al., 2003).

Greater understanding of PCD in pathogenic fungi may offer a chance of exploiting the fungal death machinery to control fungal infections. Clearly identifiable differences between the death machineries of pathogens and their hosts make this a feasible task.

## THE FUNGAL PCD MACHINERY

As pointed out earlier, the complete extrinsic apoptosis pathway and major signaling components upstream of the mitochondria (intrinsic) pathway, are not found in fungal genomes. This raises the question if there are functional homologs of these proteins, which do not share sequence similarity. A number of studies showed that expression of Bcl-2 protein members triggers (e.g., Bax) or prevents (e.g., Bcl-2) PCD in fungi (Longo et al., 1997; Fröhlich and Madeo, 2000; Polcic and Forte, 2003; Barhoom and Sharon, 2007). Thus, despite the lack of Bcl-2 homologs, proteins of the Bcl-2 family are recognized in fungi and specifically activate (pro-apoptotic members) or block (anti-apoptotic members) PCD. Studies in *B. cinerea* revealed a number of proteins that interact with the human Bcl-2 protein and might mediate the effect of this protein in the fungus. Moreover, a yeast two-hybrid screen of a *B. cinerea* expression library that was performed with some of these candidates led to identification of proteins that interact with the same Bcl-2-interacting proteins (Oren-Young and Sharon, unpublished results).

Filamentous fungi have larger genomes and more complex development programs compared to *S. cerevisiae*. It is therefore intuitive to assume that PCD pathways in filamentous species will include a larger number of proteins and would be more complex compared with *S. cerevisiae.* Indeed, a few homologs of animal apoptotic proteins that are not found in *S. cerevisiae* can be identified in genomes of filamentous species using a simple BLAST search. Some processes, such as the HI response are restricted to filamentous species and therefore genes that are involved in regulation of these processes are not present in unicellular species. More than 50 putative human and mouse apoptosis-associated genes that are not found in *S. cerevisiae* were described in *Aspergillus* and represent a potentially filamentous-specific PCD regulators (Fedorova et al., 2005). In addition to protein homologs of components of the metazoan apoptotic machinery, this list includes many fungal-specific genes, such as het loci, and species-specific protein families. Functional analyses were performed only on a small number of candidates, and therefore it is unclear how many proteins on this list are true regulators of PCD.

*Neurospora crassa HET-C2* is probably the best characterized HI gene. *HET-C2* orthologs were identified in genomes of all filamentous species and are also present in many animals and plants, but they are not found in *S. cerevisiae* or in the fission yeast *Schizosaccharomyces pombe.* The high level of conservation among Het-C2 family members is consistent with the important role of these proteins in glycosphingolipid and sphingosine metabolism, and possibly in regulation of cellular stress responses. Het-C2 shows significant similarity to human Gltp (Rao et al., 2004) and *A. thaliana* Acd11 (Brodersen et al., 2002), which catalyze intermembrane transfer of glycosphingolipids and sphingosines, respectively. *P. anserina* Het-C2 was proposed to act as a glycolipid metabolite sensor in addition to its role in glycolipid transfer, regulation of ascospore maturation, and triggering of HI (Saupe et al., 1994; Mattjus et al., 2002). The high level of sequence conservation in this family, suggests that the role of Het-C2 orthologs in *Aspergilli* PCD is likely similar.

Interestingly, many of the identified PCD-related proteins from *Aspergillus*, such as Amid, Bir1, HtrA, and CulA, are more similar to their human counterparts than to the yeast homologs. Moreover, a small number of animal apoptotic proteins, including PARP, have homologs in filamentous fungi but are not found in *S. cerevisiae*. PARPs catalyze the NAD(+)-dependent modification of proteins with poly (ADP-ribose), which play key roles in a plethora of processes including DNA repair, tumor progression, and aging. PARP is one of the known target proteins inactivated by caspase degradation in animal cells (Schlegel et al., 1996). *A. nidulans* PARP-like protein is broken down by caspase activity during sporulation-induced PCD (Thrane et al., 2004). *P. anserina* genome encodes a single protein with a PARP catalytic domain. Over-expression of the gene caused increased sensitivity to apoptosis inducers, impaired growth and pigmentation, sterility, and a shorter lifespan (Müller-Ohldach et al., 2011).

The availability of a large number of fungal genomes provides new opportunities to search for additional PCD-associated fungal genes. In many cases, homology with the entire animal ortholog is rather low or restricted to a specific domain and hence simple BLAST searches might not be sensitive enough to recognize the homology. In order to obtain a deeper coverage of the putative fungal PCD orthologs, a computer-guided approach was developed, which enables automatic searches of all available fungal genomes for presence of homologs of apoptotic proteins or domains (Shlezinger et al., 2011a). Using this approach, it is possible to identify all the fungal genes that are putative homologs of known apoptotic genes or that contain a putative apoptotic domain. Searches conducted with this program revealed that except for BIR, all other conserved apoptotic domains were absent from fungal genomes, including Bcl-2 homology (BH) domains (BH1–4), caspase recruitment domain (CARD), cellular apoptosis-susceptibility (CAS) protein, death domain (DD), death effector domain (DED), CIDE [cell death-inducing DNA fragmentation factor 45 kDa *α* (DFFA)-like effector], or death receptors. Likewise, homologs of many central apoptosis regulators, such as P53, Flip, Smac/Diablo, Apaf1, and even caspases are not readily found in fungi. It should be noted that putative homologs for some of these proteins have been reported in filamentous species, including P53 (Katz et al., 2006) and PARP (Fuchs and Steller, 2011). However in most instances homology centers around parts of the proteins that are associated with general functions, such as protein interaction, while the domain known to mediate apoptosis is usually absent. A unique exception is the *S. cerevisiae* Bxi1, a homolog of the human lifeguard 4 protein. Similar to all members of the lifeguard family of proteins, Bxi1 contains a Bax inhibitor 1 (BI-1)-like domain, and therefore was assumed to represent a yeast homolog of BI-1 (Chae et al., 2003; Cebulski et al., 2011). However, recent work has shown that this protein contains a BH3-like signature at the carboxy part of the protein. Remarkably, functional analyses confirmed a pro-apoptotic activity in these residues (Buttner et al., 2011). Search of fungal genomes using the domain-centered approach revealed a single homolog of lifeguard 4/Bxi1 in all fungi. However, in filamentous species members of the subkingdom Dikarya (Ascomycetes and Basidiomycetes), a true homolog of plant and human BI-1 was also found (Goldfinger and Sharon, unpublished). These new findings indicate that additional “missing” fungal homologs of animal apoptotic proteins and domains might be found using more robust bioinformatic approaches.

## SUMMARY

The realization in the early 1990s that yeast cells contain a suicide mechanism led to intense research of PCD in *S. cerevisiae*. The PCD response was characterized in great detail, *S. cerevisiae* homologs of mammalian apoptotic genes were identified, and the relevant proteins analyzed. Based on these studies it is now generally accepted that yeast cells contain a PCD machinery, which resembles the animal apoptosis machinery. Studies of PCD in additional fungi lagged behind the work in budding yeasts, and a more intense research was initiated only in the past decade. As expected, the machinery is similar to the one found in *S. cerevisiae*, however some differences were also revealed. Most significantly, it was realized that PCD is important for fungal pathogenicity and multicellular-level development. Furthermore, filamentous species contain more PCD-related genes, including a few homologs of animal apoptosis proteins, which are absent in yeasts, and some that are fungal specific, such as the HI proteins encoding genes. The expansion of the research to additional species also led to better mapping of apoptosis networks in fungi. Using robust bioinformatics, it was possible to not only identify more components of the PCD apparatus in fungi, but also to exclusively show what parts of the animal machinery are missing or significantly altered. From such analyses it is now clear that the entire death receptors-mediated extrinsic pathway is missing in the fungal kingdom. Further, the main regulators of the intrinsic pathway that are responsible to initiate mitochondria-related apoptosis also seem to be largely absent in fungi. These discoveries put fungal PCD in a new light; while the pioneering studies in *S. cerevisiae* uncovered the presence of PCD machinery that is highly similar to animal apoptosis, the expansion of the research to additional fungal species shows that the molecular machinery bears significant differences compared with the animal apoptotic machinery. These differences probably also reflect differences in the execution and role of PCD in fungi compared to animals. We expect that research of fungal PCD will intensify and extend to an even wider range of species, leading to a deeper understanding of the regulation of this process and the physiological roles that it has in fungal life cycles.

## Conflict of Interest Statement

The authors declare that the research was conducted in the absence of any commercial or financial relationships that could be construed as a potential conflict of interest.
